# Follicle stimulating hormone promotes production of renin through its receptor in juxtaglomerular cells of kidney

**DOI:** 10.1186/s13098-022-00816-x

**Published:** 2022-05-03

**Authors:** Zhen Yu, Jing Yang, Wen-Jie Huang, Tao Zhang, Xiao-Min Li, Wei Zhao, Xiao-Yong Li, Yong-Chao Lu

**Affiliations:** 1 Department of Reproductive Endocrinology, Women’s Hospital, Zhejiang University School of Medicine, No. 1 Xueshi Road, Hangzhou, 310006 Zhejiang China; 2 Department of Gynecology, Shangyu People’s Hospital of Shaoxing, Shangyu, Shaoxing, 312300 Zhejiang China; 3 Department of Assisted Reproduction, Shanghai Ninth People’s Hospital, Shanghai Jiaotong University School of Medicine, Shanghai, 200011 China

**Keywords:** FSHR, Juxtaglomerular cell, Renin

## Abstract

**Background:**

Post-menopausal hypertension has been attributed solely to declining estrogen levels. The purpose of the research is to elucidate the mechanism by which follicle stimulating hormone(FSH) increases renin production involved in the regulation of blood pressure.

**Methods:**

The expression of follicle stimulating hormone receptors (FSHRs) in renal juxtaglomerular cells and a As4.1 juxtaglomerular mouse cell line was evaluated. We established a mouse model by ovariectomy (OVX). Ovariectomized mice were treated with gonadotropin-releasing hormone agonist (GnRHa) (OVX + GnRHa). Ovariectomized mice initially received physiological doses of estrogen and were then injected with recombinant FSH (OVX + E + FSH).

**Results:**

We found that FSHR was expressed in mouse renal juxtaglomerular cells labeled by renin antibody and in As4.1 cells. FSH promoted renin synthesis via Gsα-coupled FSHRs that activated protein kinase A, cyclic adenosine monophosphate(cAMP) response element-binding protein, extracellular signal-regulated kinase (Erk1/2), Protein kinase B(AKT), and c-Jun N-terminal kinase signaling pathways in As4.1 cells. We found increased serum FSH levels in the ovariectomized mouse with concurrent increases in renin, angiotensin II, heart rate (HR), systolic blood pressure (SBP), diastolic blood pressure (DBP), and mean arterial blood pressure (MAP). Additionally, increases in serum renin, angiotensin II, HR, SBP, DBP, and MAP were reduced by the additional injection of GnRHa. Exogenous FSH administration completely reversed decreases in renin, angiotensin II, HR, SBP, DBP, and MAP even in mice that received physiological doses of estrogen to maintain normal estradiol levels.

**Conclusions:**

Elevated FSH stimulates renin production involving a mechanism that may be relevant to the expression of FSH receptors in renal juxtaglomerular cells.

**Supplementary Information:**

The online version contains supplementary material available at 10.1186/s13098-022-00816-x.

## Introduction

Hypertension is one of the leading risk factors for cardiovascular disease [1, 2]. Aging in both men and women is characterized by increases in blood pressure (BP) [1–4]. However, age-related increases are more rapid in women than in men [4–7], and the prevalence of hypertension in postmenopausal women is higher than that in men. In the United States, > 75% of women over 60 years old are hypertensive [5, 6]. A study showed that the percentage of individuals with uncontrolled BP was 50.8 ± 2.1% in men and 55.9 ± 1.5% in women, although women were more likely to have their BPs measured within the previous 6 months [7]. Despite this, the study also showed that women were more likely to have poorly controlled hypertension, although drugs used to treat hypertension were similar in both sexes [7]. Thus, though antihypertensive methods are similar between men and women, and women are more likely to have their BPs measured, hypertension may be less well controlled in women. This suggests that perhaps the mechanisms responsible for hypertension in women may differ from those in men.

Follicle-stimulating hormone (FSH), a glycoprotein hormone derived from the pituitary, is involved in reproductive events, such as gonadal and germ cell development, as well as in sex hormone production [8]. Circulating FSH levels also undergo dramatic changes with age because of the loss of negative feedback from inhibin, as well as estrogens, in aging females [9]. Many studies have shown that activation of the renin-angiotensin system (RAS) may be involved in BP increases in postmenopausal women [10]. For example, postmenopausal women exhibit increases in plasma renin activity compared to their premenopausal counterparts [10, 11]. Interestingly, our primary study has detected FSHR in renal juxtaglomerular cells, suggesting a possible role for FSH in promoting renin secretion in postmenopausal hypertension.

The possible role of FSH and FSHR in dysfunction of the renin–angiotensin system that may be involved in BP increases in postmenopausal women has not been investigated. Considering the observed correlation between high circulating FSH levels and activation of the renin–angiotensin system in postmenopausal women, we hypothesize that FSHR might be expressed in human renal juxtaglomerular cells and that high circulating FSH levels might modulate juxtaglomerular cell functions during aging. Therefore, we performed this study to test this hypothesis.

## Materials and methods

### Cell culture and treatment

As4.1 cells (ATCC, Manassas, VA, USA), an immortalized renin-containing mouse renal tumor cell line, were used in this study. Cells were cultured in complete DMEM medium (Invitrogen, Carlsbad, CA, USA) supplemented with 10% fetal bovine serum (Invitrogen), 1 IU/mL penicillin (Invitrogen), and 100 µg/mL streptomycin (Invitrogen).

### Immunofluorescence analysis

Mouse kidney tissues were paraffin-embedded and then sectioned at 4 μm. The As4.1 cells were seeded and grown on coverslips, and then fixed with 4% paraformaldehyde (Sigma, (Sigma, St. Louis, MO, USA) at room temperature for 1 h. The samples were permeabilized with 0.1% Triton X-100 (Sigma) in phosphate buffered saline for 20 min. Antibody staining was performed using standard protocols as described previously [12]. Antibodies used in experiments are described in Additional file 1: Table S1. Nuclei were counterstained with DAPI for 20 min. Finally, images were analyzed under a fluorescence microscope (Olympus, Tokyo, Japan).

### RT-PCR and quantitative real-time PCR analysis

Total RNA was extracted using TRIZOL Reagent (Takara, Tokyo, Japan). A 10 µg sample of total RNA was reverse transcribed into cDNA with a Prime Script™ RT reagent Kit (Takara). One microliter of cDNA was used for quantitative real-time (qRT)–PCR. PCR cycling conditions were as previously described [12] and performed in a thermal cycler (S1000™) (Bio-Rad, Foster, CA, USA). Mouse GAPDH was used as the internal control. The qRT–PCR was performed using a SYBR^→^Premix ExTaq™ reagent Kit (Takara) and carried out in an Applied Biosystems 7500 Fast PCR system (ABI, Carlsbad, CA, USA). Primer sequences are listed in Additional file 1: Table S2. Data were analyzed using the comparative threshold cycle (CT) method [13].

### Western blotting analysis

Samples were lysed with radioimmunoprecipitation buffer. Whole cell lysates were obtained by centrifugation at 15,000×*g* for 30 min at 4 °C. After the protein concentration was determined by Bradford assay (Bio-Rad Laboratories, Hercules, CA, USA), whole cell lysates containing equal amounts of protein (50 µg/lane) were separated on a 10% SDS-polyacrylamide gel and transferred to nitrocellulose membranes. Immunoblots were performed following standard procedures as described previously [12]. The antibodies used were listed in Additional file 1: Table S1. The signals were detected with enhanced chemiluminescence detection reagent (Amersham, Piscataway, NJ, USA).

### Cell transfection

Small interfering (si) RNA transfection was performed according to the manufacturer’s protocol. Briefly, 5 µL of Lipofectamine 2000 (Thermo Fisher Scientific, Shanghai, China), 20 pM of scrambled RNA (sc-44230; Santa Cruz Biotechnology, Santa Cruz, CA, USA), FSHR siRNA (sc-35415, Santa Cruz Biotechnology), and thyroid-stimulating hormone receptor (TSHR) siRNA (sc-36754, Santa Cruz Biotechnology) were diluted separately in 250 µL of serum-free Opti-MEM (Thermo Fisher Scientific) and incubated at room temperature for 5 min. The diluted scrambled RNA/siRNA pools were then mixed with diluted Lipofectamine 2000, and incubated at room temperature for 20 min. Such scrambled RNA/siRNA-Lipofectamine 2000 complexes were added to the cell cultures. Cells were incubated at 37 °C for 6 h to recover. The cDNA of mouse FSHR was cloned into pcDNA3.1 (Thermo Fisher Scientific) at the EcoRI site. Two micrograms of pcDNAhFSHR were transfected into As4.1 cells by Lipofectamine 2000. Cells stably transfected with pcDNAhFSHR were selected by selective medium that included G418 (Thermo Fisher Scientific). Individual colonies resistant to G418 and overexpressing FSHR were obtained 20 days later.

### MTT assay

To determine the effect of FSH on the proliferation of As4.1 cells, 4 × 10^3^ cells were treated with different concentrations of FSH (Gonal-F; Serono, Geneva, Switzerland; 0, 3, 10, 30, 100, and 300 ng/mL) following the manufacturer’s protocol in a 96-well plate. A (3-(4, 5-dimethylthiazol-2-yl)-2, 5-diphenyl-tetrazoliumbromide) (MTT) assay was performed 24 h, 48 h, 72 h, and 94 h post-treatment. The absorbances of samples were measured with a spectrophotometer reader at 490 nm. Each assay was performed in triplicate and repeated three times independently.

### Intracellular cAMP assay

As4.1 cells were treated with different concentrations of FSH (0, 3, 10, 30, 100, 300 ng/mL) for 15 min. Cyclic AMP degradation was inhibited by the addition of 100 µM isobutylmethylxanthine. After treatment, cells were lysed with 0.1 M HCl and centrifuged at 1000×*g* for 10 min to remove cell debris. A cAMP assay was performed by enzyme immunoassay (EIA) following the instructions of the EIA kit (901−066; Assay Designs, Ann Arbor, MI, USA).

### Radioimmunoassay

Medium supernatants and blood samples were collected to estimate FSH, LH, estradiol, renin, and angiotensin II in mouse serum by radioimmunoassay. Serum samples were separated by standard procedures and stored at −20 °C for subsequent analysis. FSH, LH, estradiol, renin, and angiotensin II were subjected to radioimmunoassay by double-antibody precipitation methods [14]. Measurements were carried out in duplicate. Samples and standards (100 µL) were preincubated overnight at 4 °C with primary antibody (100 µL). Tracer (100 µL; iodinated FSH, LH, estradiol, renin, and angiotensin II; 12,000–15,000 cpm) was added to each tube, and the tubes were incubated for another 24 h at 4 °C. Secondary antibody (500 µL) was added and incubated for 0.5 h; water (500 µL) was then added. Bound and free tracers were separated by centrifugation (1600 g) for 5 min. The supernatant containing unbound tracer was aspirated using a vacuum pump; the bound tracer in the precipitate was counted using a gamma counter.

### Animals

Mice were obtained from the laboratory animal service center of Zhejiang University. The care and use procedures for mice were in accordance with the Institutional Guide for Laboratory Animals established by the Animal Care and Use Committee (ACUC) and were approved by the ACUC of Zhejiang University School of Medicine (Ethical Approval Number: 12,320). C57BL/6 mice (8 weeks old with body weights of 18–20 g) were housed under a 12/12-h light/dark cycle at 25 ± 0.5 °C and 50–60% humidity and were fed *ad libitum* with a standard diet and water. All mice were divided into four groups (n = 10 per group): (i) sham operated, (ii) OVX, (ii) OVX + GnRHa, and (iii) OVX + estrogen + FSH. In the OVX group, the mice were gonadectomized to mimic high serum FSH levels in aging populations. In the OVX + GnRHa group, gonadectomized mice were treated with GnRHa (0.5 µg/day, triptorelin acetate; Beaufour-Ipsen, Tianjin, China) by intraperitoneal injection for 8 weeks to inhibit pituitary gonadotropin secretion. In the OVX + estrogen + FSH group, castrated mice received physiological doses of estrogen in the form of daily subcutaneous injections of 0.72 mg 17β-estradiol/kg [15] (Sigma) and simultaneously were injected with recombinant FSH (0.15 IU/day; Gonal-F; Serono) by intraperitoneal injection for 8 weeks. The sham for each group received the same volume saline injection as control. All mice were maintained for 14 days before BP, endocrine levels, and renin concentrations were measured.

### SBP and DBP measurements

SBP and DBP were non-invasively measured three times a day for three days in mice of the sham and operation groups 28 days after surgery using a computerized tail-cuff method (Softron BP98A; Softron Co., Ltd., Tokyo, Japan) as described previously [16]. The data were derived from an average of 10–20 measurements per mouse at each time point.

### Statistical analysis

Data were presented as the mean ± standard error (SEM). A two-tailed unpaired Student’s *t*-test was used for comparisons between two groups. A statistical analysis was done using One-way analysis of variance (ANOVA) and Tukey HSD test for multi-comparison. The SPSS 22.0 version for Windows (SPSS Inc., Chicago, IL, USA) was used for statistical analysis. A probability of *P* < 0.05 was considered statistically significant.

## Results

### Expression of FSHR in juxtaglomerular cells of kidney

**Fig. 1:**
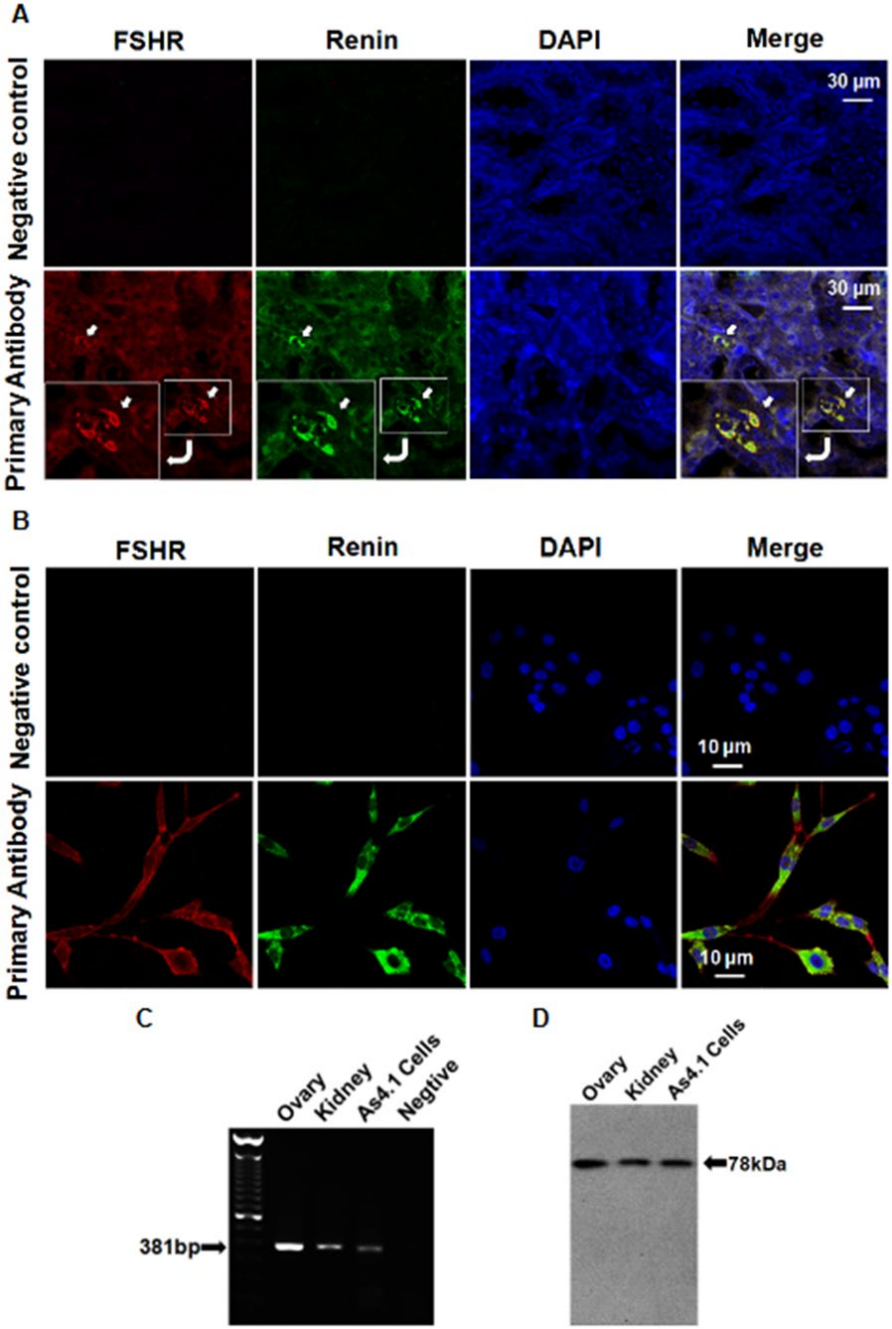
Expression and location of FSHR in mouse kidney and JG cells.** A** Confocal images of kidney sections stained with FSHR (red), renin (green), or nucleus (blue) from mouse. (×200). In negative controls PBS replaced the primary antibody. Scale bar: 30 μm. **B** Immunofluorescent staining of FSHR in the As4.1 cells. (×400). In negative controls PBS replaced the primary antibody. Scale bar: 10 μm. **C** RT-PCR analysis shows that a 381-bp band representing FSHR mRNA was detected in mouse ovary (positive control), kidney and As4.1 cells, but not in negative control where cDNA was replaced by water. **D** Western blotting analysis showed that a 78-kDa band representing FSHR protein was detected in mouse ovary (positive control), kidney and As4.1 cells

The expression of FSHR (red) and renin (green) in the mouse kidney was detected by confocal double immunofluorescence. FSHR was mainly localized in the membrane of juxtaglomerular cells of the mouse kidney (Fig. 1A) and the membrane of As4.1cells (Fig. 1B**)**. RT–PCR and western blotting analyses confirmed the expression of FSHR mRNA and protein in mouse kidney and As4.1 cells (Fig. 1C and D).

### FSH promotes renin expression and production

**Fig. 2:**
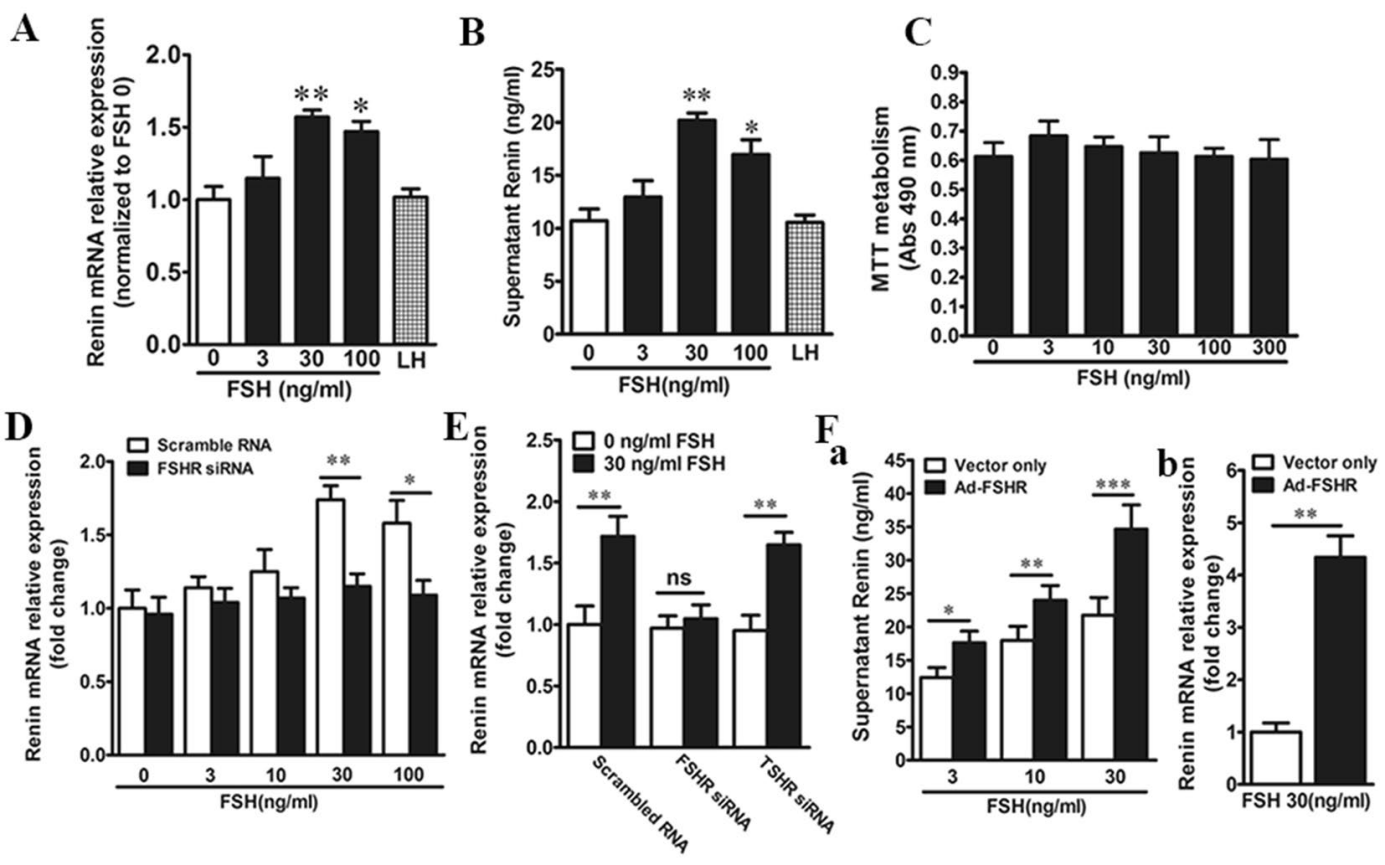
FSH Stimulates As4.1 cells to produce rennin.** A** Quantitative real-time PCR shows the effect of FSH or LH (100 ng/ml) on rennin mRNA expression in 2-day cultures from As4.1 cells. Data are presented as mean ± SEM; n = 5; *P < 0.05, **P < 0.01, compared with the 0ng/ml FSH treatment. *P*-values were 0.0073, 0.0386, respectively. **B** The in vitro culture shows the effect of FSH or LH (100 ng/ml) on the production of renin as the supernatant renin concentration in As4.1 cells. Data are presented as mean ± SEM; n = 5; *P < 0.05, **P < 0.01, compared with the 0ng/ml FSH treatment. *P*-values were 0.0067, 0.0413, respectively. **C** FSH has no effect on the proliferation of As4.1 cells. Mean absorbance (Abs, ±SEM) at 490 nm. Data are presented as mean ± SEM; n = 5; compared with the 0ng/ml FSH treatment. **D** Effect of FSH on rennin mRNA expression in As4.1 cells after FSHR knockdown by siRNA transfection using quantitative real-time PCR. Data are presented as mean ± SEM; n = 5; *P < 0.05, **P < 0.01, compared with the corresponding controls. *P*-values were 0.0047, 0.0334, respectively. **E** Effect of FSH on rennin mRNA expression in scrambled RNA trasfection (control), FSHR siRNA trasfection or TSHR siRNA trasfection in As4.1 cells. Data are presented as mean ± SEM; n = 5; **P < 0.01, compared with the corresponding controls. *P*-values were 0.0068, 0.0052, respectively. **F** Effects of FSH on rennin concentration in supernatant of medium by invitro culture (**Fa**) and rennin mRNA by quantitative real-time PCR (**Fb**) in As4.1 cells infected with adenovirus containing the mouse FSHR gene or empty vector. Data are presented as mean ± SEM. n = 5; *P < 0.05, **P < 0.01, ***P < 0.001, compared with the corresponding controls. *P*-values for panel **Fa** were 0.04065, 0.00776, 0.00083, respectively. *P*-values for panel **Fb** was 0.0038

As4.1 cells were stimulated in a concentration-dependent manner with significant effects on renin expression at 30 and 100 ng/mL FSH (*P* < 0.05) (Fig. 2A). 30 and 100 ng/mL FSH similarly stimulated the production of renin as shown by the supernatant renin concentration in As4.1 cells cultures (*P* < 0.05) (Fig. 2B). LH (100 ng/mL) did not affect renin expression in As4.1 cell cultures (*P* > 0.05) (Fig. 2A and B). Overall, the *in vitro* results are in keeping with the expression of FSHR in juxtaglomerular cells. We attempted to distinguish the effects of FSH on proliferation versus renin synthesis in As4.1 cells. We found that FSH did not affect As4.1 cell proliferation (*P* > 0.05) (Fig. 2C) but stimulated the expression of renin at the concentration of 30 and 100 ng/ml (*P* < 0.05) (Fig. 2D). We next sought to eliminate any interaction between FSH and TSHR. FSH stimulated renin expression in TSHR knockdown but not FSHR knockdown cultures (Fig. 2E), ruling out a role for TSHR. As a further control, we overexpressed FSHR in As4.1 cells and found evidence of augmented supernatant renin concentration and renin mRNA expression compared with vector-only controls (Fig. 2Fa and b). Together, these data indicate that the FSH effects are mediated solely through the FSHR in As4.1 cells.

### High levels of FSH affect renin elevation and BP increase in mice

**Fig. 3:**
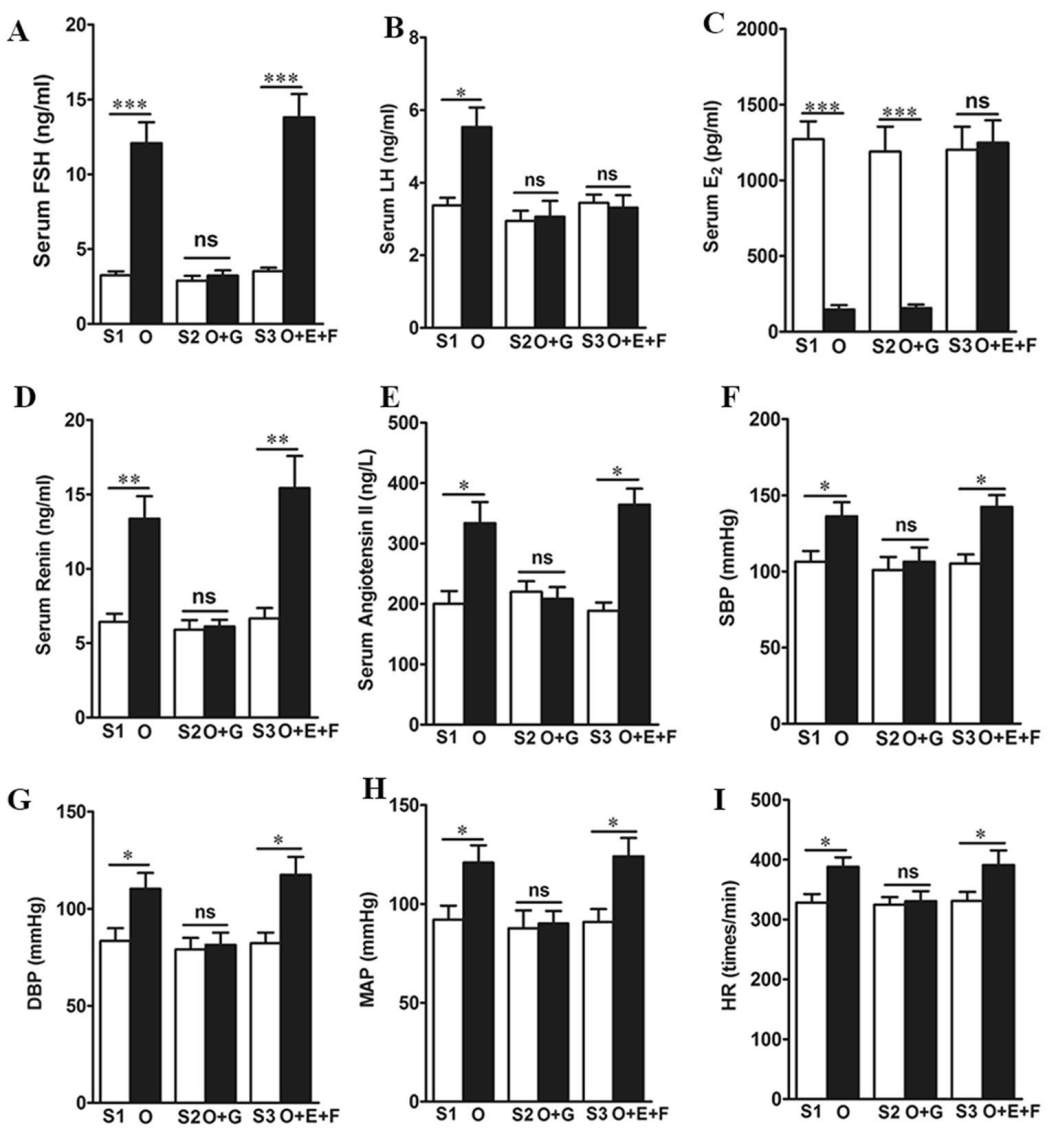
High level FSH, not low estrogen, increased the mouse serum rennin levels and BP. The serum FSH (**A**), LH (**B**), E_2_ (**C**), rennin (**D**) and angiotensin (**E**) in different treatment groups. The SBP (**F**), DBP (**G**), MAP (**H**) and HR (**I**) in different treatment groups. Data are presented as mean ± SEM. 10 mice/group, *P < 0.05, **P < 0.01, ***P < 0.001, compared with the corresponding controls. ns, not significant. S, sham group; O, OVX (ovariectomized mice); O + G (ovariectomized mice injected with GnRHa); O + E + F (ovariectomized mice injected with estrogen and FSH). *P*-values for panel **A** were 0.00026, 0.00018, respectively. *P*-value for panel **B** was 0.0318. *P*-values for panel **C** were 0.00054, 0.00032, respectively. *P*-values for panel **D** were 0.0065, 0.0047, respectively. *P*-values for panel **E** were 0.0266, 0.0187, respectively. *P*-values for panel **F** were 0.0412, 0.0375, respectively. *P*-values for panel **G** were 0.0306, 0.0285, respectively. *P*-values for panel **H** were 0.0446, 0.0349, respectively. *P*-values for panel **I** were 0.0415, 0.0307, respectively

In the three animal models (ovariectomy (OVX), OVX+GnRHa injection, OVX+ Estradiol (E)+ follicle stimulating hormone (FSH) injection) used in this study, we found increased serum FSH and luteinizing hormone (LH), and reduced estradiol levels in OVX animals (*P* < 0.05) (Fig. 3**A**, **B** and **C**), with increases in renin, angiotensin II, the heart rate (HR), SBP, DBP, and mean arterial blood pressure (MAP) (*P* < 0.05) (Fig. 3**D**, **E**, **F**, **G**, **H** and **I**). Interestingly, the increases in serum renin, angiotensin II, SBP, DBP, MAP, and HR, were obtained by additional gonadotropin-releasing hormone agonist (GnRHa) injection (Fig. 3**D**, **E**, **F**, **G**, **H** and **I**). In addition, we found that in the OVX mouse, combined with an E injection treatment with an additional FSH injection showed significant increases in renin, angiotensin II, SBP, DBP, MAP and HR when serum FSH increased (*P* < 0.05) (Fig. 3**D**, **E**, **F**, **G**, **H** and **I**). Together, these data indicate that an increased high level of serum renin, angiotensin, as well as increased BP are related to a high level of FSH in serum.

### FSH activates MEK/Erk and Akt signaling pathways via G_sα_ to promote renin production

**Fig. 4:**
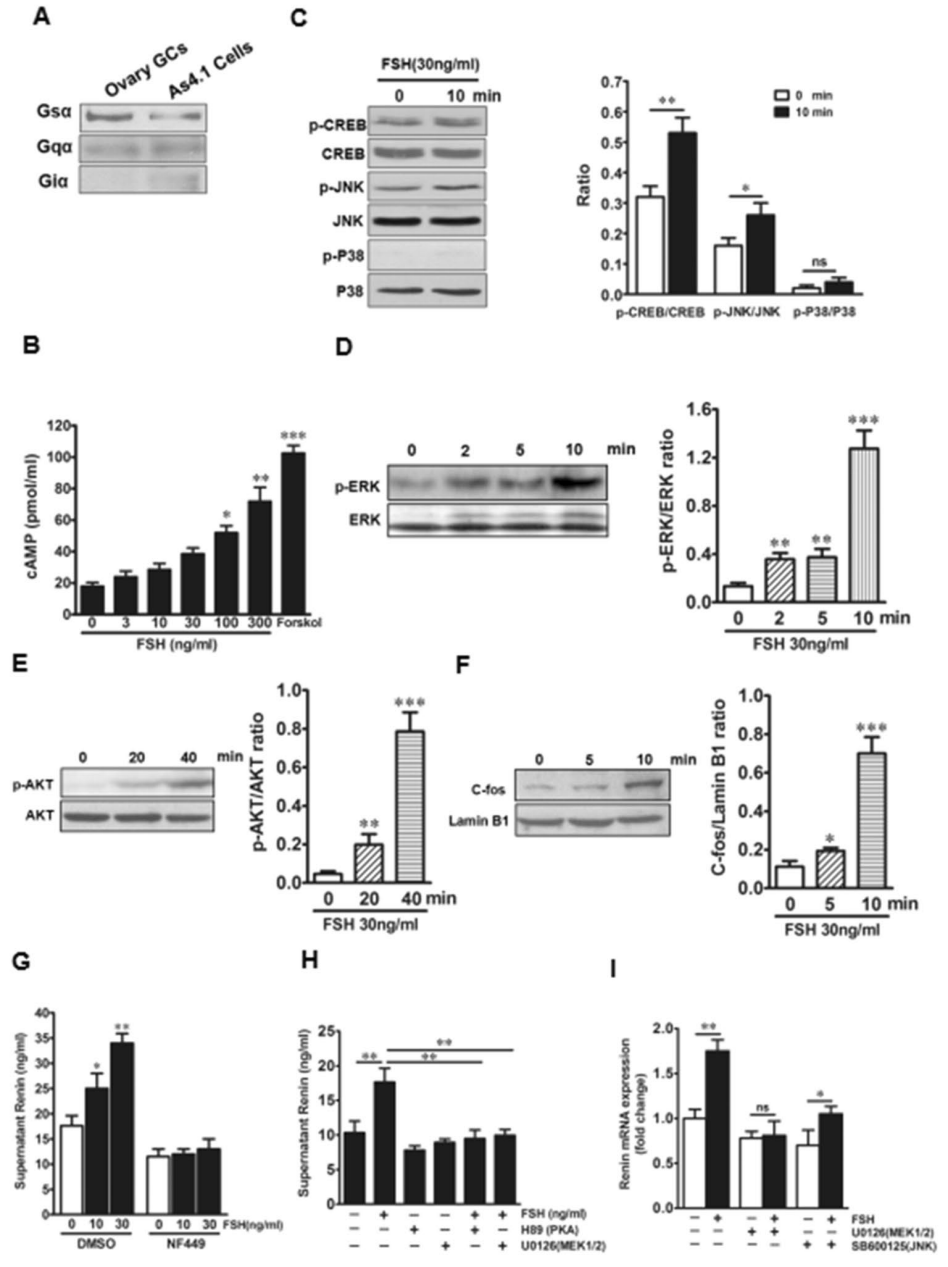
FSH activates Erk, Akt, and CREB via Gsα to produce rennin in As4.1 cells.** A** Western blotting results shows Gsα isoforms is expressed in As4.1 cells. **B** Effect of FSH and forskolin (Forskol, 10 mM) on cAMP levels in As4.1 cells cultures. Data are presented as mean ± SEM; n = 5; *P < 0.05, **P < 0.01, ***P < 0.001, compared with the 0ng/ml FSH treatment. *P*-values were 0.03228, 0.00574, 0.00072 respectively. **C**–**F** Western blots of phosphorylated (p-) Erk1/2, Akt, CREB, JNK, or p38 levels or nuclear c-Fos levels in As4.1 cells in response to FSH. Data are presented as mean ± SEM; n = 5; *P < 0.05, **P < 0.01, ***P < 0.001, compared with the 0ng/ml FSH treatment. *P*-values for panel **C** were 0.0069, 0.0297, respectively. *P*-values for panel **D** were 0.00533, 0.00229, 0.00076, respectively. *P*-values for panel **E** were 0.00178, 0.00043, respectively. *P*-values for panel **F** were 0.03985, 0.00085, respectively. **G** Effect of FSH on rennin concentration in the supernatant of As4.1 cells cultures after Gsα was inhibited by NF449 (100ng/mL). Data are presented as mean ± SEM; n = 5; *P < 0.05, **P < 0.01, compared with the 0ng/ml FSH treatment. *P*-values were 0.0414, 0.0075, respectively. **H** Effect of PKA inhibitors H89 (30 µM) and MEK1/2 inhibitors U0126 (0.5 mM) on rennin concentration in the supernatant of As4.1 cells cultures in response to 30 ng/ml FSH treatment. Data are presented as mean ± SEM; n = 5; **P < 0.01, compared with the corresponding controls. *P*-values were 0.0082, 0.0061, 0.0047, respectively. **I** Quantitative real-time PCR showing the effect of the MEK1/2 inhibitors U0126 (0.5 mM) or JNK inhibitors SB600125 (0.5 mM) on rennin mRNA expression in 48-hour cultures. Data are presented as mean ± SEM; n = 5; *P < 0.05, **P < 0.01, ***P < 0.001, compared with the corresponding controls. *P*-values were 0.0056, 0.0309, respectively

FSHR in renin synthesis couples with the stimulatory G protein (G_sα_). The mouse As4.1 cells expressed G_sα_ (Fig. 4 A). Consistent with the activation of G_sα_, FSH triggered a concentration-dependent increase in cyclic adenosine monophosphate (cAMP) levels, with the highest level after treatment with forskolin, an agonist of G_sα_ (Fig. 4B). We then examined whether FSH stimulated CREB, MAP kinase (MEK/Erk, JNK, and p38), and AKT pathways. FSH stimulated CREB, Erk1/2, AKT, and JNK phosphorylation within 10 min but did not phosphorylate p38 in As4.1 cells (Fig. 4C and D). In addition, FSH rapidly enhanced the phosphorylation of Erk1/2 and Akt as well as the expression of c-Fos in As4.1 cells (Fig. 4D–F). This suggests that Erk1/2 and c-Fos are downstream of G_sα_. Consistent with this, FSH-induced increases in renin production were abrogated by NF449, a special inhibitor of G_sα_ in As4.1 cells (Fig. 4G). We next examined the effects of each downstream signaling pathway in mediating the functions of FSH. The MEK1/2 inhibitor, U0126, and the PKA inhibitor, H89, abrogated the FSH-induced increase in supernatant renin concentration (Fig. 4H). In the presence of the JNK inhibitor, SB600125, FSH still significantly increased renin expression (*P <* 0.05), although the effect was attenuated (Fig. 4I). This attenuation was in concordance with the minimal induction of JNK phosphorylation by FSH (Fig. 4C). Together, the results established that CREB, MEK/Erk, AKT, and JNK pathways contributed to the effects of FSH on renin synthesis.

## Discussion

This study determined whether FSH directly affects the secretion of renin. We showed that FSH stimulated FSHR localized in juxtaglomerular cells to produce renin *in vitro* and *in vivo*. These actions are exerted via a Gsα-coupled FSHR that triggers MEK/Erk and Akt signaling pathways. The increased high level of serum renin and angiotensin as well as increased BP are related to the high level of FSH in serum of animal models, even in the presence of normal estrogen levels. This indicates an estrogen-independent action of FSH on BP regulation.

FSH levels are drastically increased during aging [9], especially in postmenopausal females, but the contributions of FSH to aging processes are remain unclear. Several physiological systems have been implicated in clinical studies. For example, postmenopausal women exhibit increases in plasma renin activity compared to their premenopausal counterparts [10, 17]. This suggests that increased FSH levels are correlated with known cardiovascular disease (CVD) risk factors and reveals the critical roles of FSH in renin activity. Our present study demonstrates that FSHRs are expressed in the juxtaglomerular cells of the mouse kidney, and a mouse juxtaglomerular cell line. This suggests a possible role for high FSH levels in the serum renin increase observed in aging subjects.

In the adult kidney, renin is synthesized by myofibroblast-like cells that are located at the medial layer of renal afferent arterioles at the entrance to the glomerular capillary network [18]. Only juxtaglomerular cells in the kidney are capable of generating active renin from prorenin, which is stored in prominent vesicles and released into the circulation upon demand [18]. Using mouse juxtaglomerular cell lines *in vitro*, we demonstrated that FSH directly stimulated juxtaglomerular cells to produce renin. In addition, FSH did not affect cell proliferation but stimulated the expression of renin. Considering the increase of supernatant renin concentration, FSH stimulated juxtaglomerular cells to release renin from the cytoplasm. FSHRs are members of the TSHR/LHR/FSHR superfamily [19]. We therefore sought to examine whether FSH displayed an agonistic action toward the TSHR. We found that FSH elicited a full renin expression response in TSHR knockdown cultures, ruling out a role for TSHRs. We further established specificity by showing enhanced renin expression in FSHR-overexpressing cultures.

We found that the FSHRs localized in juxtaglomerular cells were coupled to Gsα, the major Gs isoform to form complete G protein coupled receptor (GPCRs). FSH-induced increases in cAMP levels attest to this coupling. Whereas a similar coupling of the FSHR to Gsα has been noted in granulosa cells in the ovarian follicle [20], FSH actions in luteal granulosa and Sertoli cells are mediated via Giα [21, 22]. It has previously been reported that binding of FSH to its receptor results in adenyl cyclase activation leading to cAMP synthesis, which is associated with the activation of phosphorylated CREB [23, 24], protein kinase A, and MAP kinase pathways [21, 25]. The effects of FSH on Erk1/2, AKT, CREB activation, and c-Fos expression were all investigated in juxtaglomerular cells. Consistent with this, FSH-induced increases in renin concentration in the culture medium were abrogated by NF449, a Gsα inhibitor. This suggests that Erk1/2, CREB, AKT, and c-Fos are downstream of FSHR for the synthesis of renin in juxtaglomerular cells. We also find that FSH-induced renin synthesis is attenuated by MEK inhibitors but not by a JNK inhibitor, indicating that the MEK/Erk pathway is essential. The synthesis of renin that was increased by FSH was also abrogated by H89, a PKA inhibitor. Overall, FSH activates Erk1/2, Akt, and CREB, but not p38 and JNK, to stimulate renin synthesis in juxtaglomerular cells.

Because the primary mechanisms underlying a cardiovascular protective effect have been attributed solely to estrogen in the past [26], the direct action of FSH on renin synthesis has never been explored. Increased FSH levels in post-menopausal women likely cause age-related cardiovascular disorders. This notion is supported by the results obtained from OVX mice, which provide an experimental model of the high FSH levels observed in aging women [27]. We thus examined the *in vivo* effects of OVX, or ectogenic estrogen and FSH after OVX, on renin synthesis. Despite persistent low estradiol levels, GnRHa reversed OVX-induced changes in BP and HR in mice. Hence, high FSH and/or LH levels are possibly responsible for cardiovascular disorders in aging women [28, 29]. However, the replenishment of recombinant FSH in OVX mice that received ectogenic physiological doses of estrogen resulted in a significant increase in BP and HR, which excludes the possible effect of LH and low estrogen; this result affirmed that FSH is responsible for cardiovascular disorders. Similar with the results obtained *in vitro*, studies with OVX mice also showed that FSH was responsible for the increased serum levels of renin and angiotensin II. Moreover, FSH was implicated in increased renin synthesis *in vivo*. These results indicated the contribution of FSH to renin-related cardiovascular disorders in mice. Nevertheless, further studies should be conducted to confirm this possibility.

There were still some limitations in this study. The interaction between FSHR and Gsα has not been evaluated. Histology analysis and serum biomarkers such as urine, creatinine for kidney function have not been performed in this study. The related pathways of the involvement of FSH in a renin synthesis disorder have not been evaluated. The renin concentration of FSHRs in TSHR knockdown conditions was not investigated and whether FSH displayed an agonistic action toward the LHR has also not been evaluated.

## Conclusions

In conclusion, our findings revealed a potential role for FSH in promoting renin synthesis in humans. Our results also outlined a new molecular mechanism for age-related cardiovascular disorders in women. High levels of FSH, rather than decreased levels of sex hormones, are possibly responsible for an increase of renin in serum in aging populations of females. This study demonstrated that FSH was involved in a renin synthesis disorder. Along with high FSH levels commonly observed in aging women, FSHR might be considered a potential therapeutic target to reduce the risk of age-related cardiovascular disorder and diseases.

## Supplementary Information

**Additional file 1: Table S1.** Antibody information. **Table S2.** Nucleotide sequences of primers used for RT-PCR and quantitative real-time PCR (qPCR) (SYBR Green).

## Data Availability

All data generated or analysed during this study are included in this. Further enquiries can be directed to the corresponding author.

## Abbreviations

FSH: Follicle stimulating hormone
FSHRs: Follicle stimulating hormone receptors
OVX: Ovariectomy
HR: Heart rate
SBP: Systolic blood pressure
DBP: Diastolic blood pressure
MAP: Mean arterial blood pressure
BP: Blood pressure
RAS: Renin-angiotensin system
ACUC: Animal Care and Use Committee
SEM: Standard error
ANOVA: Analysis of variance
cAMP: Cyclic adenosine monophosphate
CVD: Cardiovascular disease
