# Supplementary material for: Follicle stimulating hormone promotes production of renin through its receptor in juxtaglomerular cells of kidney
Source: Diabetol Metab Syndr. 2022 May 3;14:65. doi: 10.1186/s13098-022-00816-x (PMC9063271; doi:10.1186/s13098-022-00816-x)
Supplement: Supplementary file 1 — Additional file 1: Table S1. Antibody information. Table S2. Nucleotide sequences of primers used for RT-PCR and quantitative real-time PCR (qPCR) (SYBR Green). [file 13098_2022_816_MOESM1_ESM.doc]

**Table S1.** Antibody information.

| **Antibody name** | **Manufacture**  **(catalogue number)** | **Manufacture (address)** | **Applications**  **(working dilution)** |
| --- | --- | --- | --- |
| anti-FSHR | Abcam (ab150557) | Cambridge, MA | IF (1:500) WB (1:1000) |
| anti-Renin | Proteintech Group (14291-1-AP) | Rosemont, IL | IF (1:200) |
| anti-Gqα | Santa Cruz (sc-365906) | Santa Cruz, CA | WB (1:200) |
| anti-Gsα | Santa Cruz (sc-135914) | Santa Cruz, CA | WB (1:200) |
| anti-Giα | Santa Cruz (sc-13533) | Santa Cruz, CA | WB (1:200) |
| anti-p-CREB | Abcam (ab10564) | Cambridge, MA | WB (1:500) |
| anti-CREB | Abcam (ab31387) | Cambridge, MA | WB (1:500) |
| anti-p-ERK | Abcam (ab223500) | Cambridge, MA | WB (1:1000) |
| anti-ERK | Abcam (ab17942) | Cambridge, MA | WB (1:500) |
| anti-p-AKT | Abcam (ab38449) | Cambridge, MA | WB (1:500) |
| anti-AKT | Abcam (ab8805) | Cambridge, MA | WB (1:500) |
| anti-p-JNK | Abcam (ab124956) | Cambridge, MA | WB (1:500) |
| anti-JNK | Abcam (ab17946) | Cambridge, MA | WB (1:1000) |
| anti-p-P38 | Abcam (ab4822) | Cambridge, MA | WB (1:500) |
| anti-P38 | Abcam (ab170099) | Cambridge, MA | WB (1:500) |
| anti-C-fos | Abcam (ab190289) | Cambridge, MA | WB (1:500) |
| anti-LaminB1 | Abcam (ab16048) | Cambridge, MA | WB (1:1000) |

**Table S2.** Nucleotide sequences of primers used for RT-PCR and quantitative real-time PCR (qPCR) (SYBR Green).

| **Target RNA** | **Forward (5’- 3’)** | **Reverse (5’- 3’)** | **Product Size** |
| --- | --- | --- | --- |
|  |  |  |  |
| mouse FSHR  (for RT-PCR) | AAACTGGAGGCGGCAAAC | GCAAAGGCGAGGTTACAC | 381 bp |
| mouse Renin  (for qPCR) | GCACCTGGCTACAGTTCACA | ATTGGGGTTCTACGGGGCT | 97 bp |
| mouse GAPDH  (for qPCR) | CCCCAGCAAGGACACTGAGCAAGAG | GCCCCTCCTGTTATTATGGGGGTC | 107 bp |
